# Abusive head trauma in small children — a single-centre experience in Finland

**DOI:** 10.1007/s00381-022-05715-0

**Published:** 2022-10-26

**Authors:** Niina Salokorpi, Juha-Jaakko Sinikumpu, Willy Serlo

**Affiliations:** 1 Department of Neurosurgery, Neurocenter, Oulu University Hospital, Oulu, Finland; 2 Unit of Pediatric Surgery and Orthopedics, Oulu University Hospital, Oulu, Finland; 3 Research Unit of Clinical Medicine, Faculty of Medicine, University of Oulu, Oulu, Finland

**Keywords:** Shaken baby syndrome, Child abuse, Brain injuries, Legal aspects, Court decisions

## Abstract

Shaken baby syndrome (SBS) is a challenging condition from both a medical and legal perspective. The path of the patients differs significantly from those with noninflicted traumas. While treating these cases, it is essential that all history, information and treatment are comprehensively documented. This article describes the investigations and interventions necessary as soon as SBS is suspected. The Oulu University Hospital protocol for suspected child abuse is described. Authors also give an overview of the SBS path in Finland from the police and prosecution’s point of view.

## Introduction

Abusive head trauma (AHT) in small children, and shaken baby syndrome (SBS) in particular, is a challenging subject both medically and legally. The path of the patients with AHT or SBS differs from all other paediatric patients with traumas. The patients are often brought to treatment with delay when their symptoms and clinical condition have already declined significantly [1, 2]. A child may be critically sick or dying at the time of admission [3, 4]. Furthermore, in more than half of the cases, there are no mentions of trauma at all on admission [1, 5, 6]. Alternatively, at most a mild, low-energy trauma is mentioned. Therefore, the diagnostic work-out in the beginning may be based on false assumptions with no screening done for abuse. If abuse is not suspected, the child may be returned to a violent home, leaving the child in high risk of reinjury and/or death [4, 7].

There are great differences in the protocols for diagnosis and medical management in emergency as well as the legal management between the countries and institutions. Legal and social aspects, among others, need special attention when treating these patients.

The purpose of this article is to describe how the primary investigations and treatment with suspected shaken baby syndrome and child abuse in general are performed at the Oulu University Hospital, Oulu, Finland, with respect of legal and child welfare aspects.

## Legislation supports prompt recognizing

National Finnish laws stress the importance and priority of recognizing, managing and keeping authorities informed promptly of all cases with suspected child abuse or neglect. The main law is the Child Welfare Act no. 417/2007, (amendments 1292/2012 and 1.1.2020), www.finlex.fi. According to its Sect. 25 (88/2020), all persons employed by or in position of trust for particular services in the society have a duty to notify child welfare authorities without delay if they, in cause of their work, discover a child for whom it is necessary to investigate the need for child welfare on account of the child’s need for care, circumstances endangering the child’s development or the child’s behaviour. This duty concerns the personnel in following services: social and health care, education, the police, fire and rescue, a reception centre for people seeking international protection and a parish or other religious communities. Furthermore, all of them must inform the police if there is any suspicion of child abuse. These obligations are superior to any laws or regulations that otherwise might cause professional confidentiality obligation. Based on this straightforward legislation, all institutions and operators within social services, health care and education have their protocols to act in cases of suspected child neglect or abuse.

## Primary investigations at appointment

A child, who is suspected to be a victim of abuse or neglect, is always admitted to the Oulu University Hospital, even if the current clinical condition would not require any treatment at a tertiary hospital. At the Oulu University Hospital, whenever there is suspicion of child abuse, a strict protocol is followed. Thorough clinical investigation is done by paediatrician or paediatric surgeon. Images are taken of all skin changes that are potentially traumatic. Comprehensive clinical investigation includes also palpation of all extremities, abdomen, back and the head. The head circumference is measured, because its increase may be the first finding in the mildest cases of SBS [5]. Leisured observation of a child aids in evaluating his/her movements of all extremities and the head. Attention is paid to the interaction and communication of the parents not only with each other but also with the child.

Laboratory tests include liver enzymes, blood coagulation status and urine sediment for latent abdominal injuries. In all patients <1 year of age the screening protocol for suspicion of abuse includes head and spinal magnetic resonance imaging (MRI), ophthalmological investigation for retinal bleeding and skeletal radiographies. Two paediatric radiologists with experience of children’s neuroradiology review the MRI images separately. If any fractures are found in radiographs, metabolic bone disorders are examined to exclude a natural reason for a fracture. The normal radiographs with intact bones are repeated 2 weeks later in order to recognize potential fractures by secondary callus formation.

It should be mentioned that according to Oulu University Hospital protocols, any bone fracture in a child, aged <1 year, is primarily seen as abuse unless an accident is proven (e.g. a traffic accident with objective witnesses).

If the work-out in the emergency department is started on wrong assumption, with no suspicion of abuse, the first investigations are based on the patient’s symptoms. Brain imaging is performed when specific symptoms are present even if trauma history is uncertain or lacking. These include seizures, symptoms of increased intracerebral pressure and loss of consciousness. If the first imaging modality is ultrasound (US) due to its better availability, the MRI is still performed as soon as it is possible.

Every time a child is admitted to in-hospital investigations and care, she/he will not be discharged without the permission of child welfare authorities. They will decide if the child has to be taken into custody. The parents are informed that there are signs of trauma found, and that the child welfare authorities will be involved. Occasionally, previously “forgotten” minor trauma will be mentioned by the parents at this stage.

## Meeting the family or guardians

The SBS, especially if the patient is in a need of an intensive care, is a dramatic and controversial condition which causes great psychological burden to the family as well as to the healthcare personnel involved in the patient’s treatment. There is only one culprit, but all caregivers are suspected and interrogated. When parents come to the hospital to see their child, they are not allowed to be alone with him/her. Furthermore, it is possible that other children in the family will be taken into custody by the authorities. For medical personnel, it may be distressing to suspect abuse, because there may be fear of making a false-positive accusation when contacting the police and child welfare authorities. Overall, physicians and other personnel in the healthcare services have to prepare themselves to meet negative and hostile reactions by the caregivers when child abuse is suspected.

## Collaboration with criminal investigation

While medical investigations and treatment are ongoing, the legal interventions must be addressed simultaneously without delay. As soon as abuse is suspected, both police and child welfare will be informed. In Finland, child welfare authorities are informed about all infants <1 year of age suffering from any fractures, and a routine screening for abuse is done, even if abuse is not suspected by clinicians. The police are the authority responsible for criminal investigation. However, in order not to lose the co-operation of the parents in medical care of the child, involving the police should not be done provokingly.

The police initiate investigations and as part of it interrogate all caregivers as soon as possible. The actions of the police depend on the certainty of abuse suspicion and severity of the injury. The information provided by the physicians is extremely important for the police investigation to be successful. Investigation when SBS or any other abusive trauma in a small child is suspected differs from any other assault investigations. This is because in many cases, there are no external signs of trauma, the victim is unable to give a statement for himself/herself, there are very rarely any witnesses and parents/caregivers statements are unreliable. Thus, investigation is initiated based on the information given by the physicians, and it is dependent on it to a great extent. A detailed statement, where the opinion of the physician is expressed clearly, is beneficial for the police. Based on this, the necessary legal actions are decided, and an investigation can be initiated. Of course should another explanation for the traumas be discovered, nobody would be accused or judged. Once all medical investigations have been performed, medical authorities will prepare a final statement which is the most important piece of evidence for the prosecution. Suspects very rarely admit to the abuse on interrogations. Furthermore, the stories told by the caregivers tend to change with time. Therefore, it is important to thoroughly question the caregivers on admission and document everything. The following questions are important: when and how the first symptoms were noticed, and what is the parents’ explanation for the findings. Also, all new previously unmentioned details or new explanations should be comprehensively documented as soon as they come to the medical personnel’s knowledge.

After the primary investigation is done by the police, the case is sent to the prosecutor for consideration of charges. The case can be closed at this stage or forwarded to court. A typical example of a case closed at this stage is a case where it is impossible to accuse one of the parents since both had equal possibility of being culprits and therefore neither could be charged. If the preliminary investigation points out a suspect, the charges can be raised, and the case can proceed to court. Usually, one of the treating physicians is invited as a witness, though sometimes a well-written medical statement is enough on its own. Unfortunately, it is still possible that nobody will be prosecuted, if no culprit could be clearly pointed out. It is worth mentioning that a caregiver’s confession of the abuse to healthcare personnel cannot be used against them in the court of law, as in Finland no one can be made to witness against himself or herself.

## Deductive consideration of likelihood

For prosecution in Finland, it is not important if the diagnostic triad of SBS (subdural haematomas, retinal haemorrhages and encephalopathy) is present in full extent. The presence of trauma in a child, with abuse considered to be the most probable or only possible explanation to it, is enough. Thus, the presence of subdural haematomas and brain contusion without retinal bleeding or fractures with the absence of any explanatory mechanisms, such as high-energy trauma (for example motor vehicle accident if findings are purely acute), is enough to suspect abusive head trauma.

According to privacy protection in Finland, there is no data available to physicians on the legal outcome of particular cases or what actions were taken by welfare. This is why there is no statistical data available. One could learn about court verdicts only from newspapers or by asking for publicly available court decisions.

## Authors’ experience in 23 years of period

The authors’ experience comprises 19 consecutive SBS cases treated during the years 1995–2018. A majority of the patients (*N*=13, 68%) were admitted to the hospital due to reasons other than trauma. Bruises were found in seven patients (37%), which is in accordance with previous findings [8]. The alleged injury pattern according to parents was, in four cases, falling from furniture or off of the caregiver’s lap. In one case, the parent claimed to be shaking the child to invigorate him. One child was involved in a traffic accident and the chronical subdural haematomas, but no acute injuries were found on the brain MRI.

The following authentic case demonstrates the controversies in both medical and legal decisions. A 3-month-old infant was admitted to the hospital directly from his christening ceremony due to fever, breathing difficulties and increased irritability. The infant was very tired, dehydrated and almost unconscious on admission.

Pulmonary infection was first suspected in the infant. However, while conventional radiographs of thorax were taken due to suspected pneumonia, three costal fractures with secondary bone healing (callus formation) were found. MRI of the brain was performed, because of suspicion of abuse. Widespread diffuse axonal injury (DAI) and subdural effusions were recognized, resulting in SBS being suspected.

**Fig. 1:**
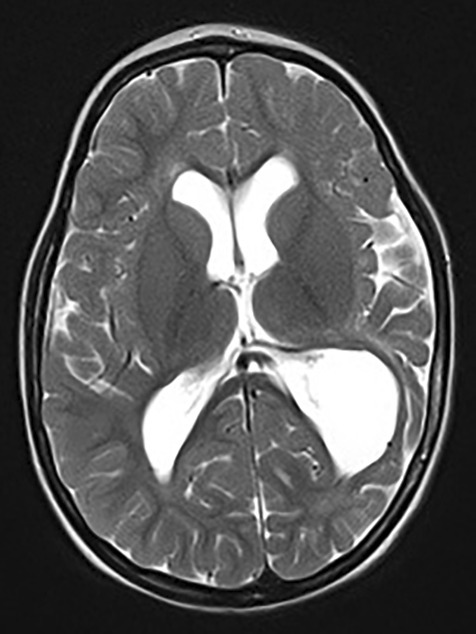
Brain MRI demonstrating atrophy of the left temporoparietal lobe 2 years after the trauma

The patient was admitted to the intensive care unit. The chronical subdural haematomas were drained. Repeated MRI scans were performed during the follow-up, and they presented progressive brain tissue atrophy (Fig. 1). Finally, after 4 weeks of treatment at the tertiary hospital, the patient was referred to a secondary level hospital for rehabilitation. Full recovery was never achieved, and the patient suffered from permanent neurological deficits.

When anamnesis was updated, it appeared that the father, a prisoner, was participating in the celebration and was seen to be shaking the child. The father was arrested and prosecuted for child abuse. Several witnesses confirmed that they had seen the father anxiously reviving the patient when he had been critically ill and weak. They stated that the child had been extraordinarily tired and seemed to have been breathing superficially. Therefore, the father was nervous and in distress and aimed to invigorate the patient by slapping and shaking him slightly. This was done with good intent according to the witnesses, resulting in the court declaring the father as innocent. The costal fractures did not affect the judgment, even though according to the medical statement they were of an older origin.

## Conclusion

As with any mild traumatic brain injury, in mild SBS, some cases probably go unnoticed. The cases with SBS detected may demonstrate just a tip of an iceberg. Authors hope that this article encourages the physicians to dare to suspect abuse and gives a good vision on what actions should be taken as soon as abuse is suspected. It highlights importance of well-conducted medical investigation and thoroughly documented anamnesis and explains why it is important to police authorities. One should not hesitate to contact police with no delay.

## Data Availability

Nothing to comment.

## Abbreviations

SBS: Shaken baby syndrome
AHT: Abusive head trauma
MRI: Magnetic resonance imaging
